# Fiber-Optic Distributed Sensing Network for Thermal Mapping of Gold Nanoparticles-Mediated Radiofrequency Ablation

**DOI:** 10.3390/bios12050352

**Published:** 2022-05-18

**Authors:** Akbota Sametova, Sabit Kurmashev, Zhannat Ashikbayeva, Aida Amantayeva, Wilfried Blanc, Timur Sh. Atabaev, Daniele Tosi

**Affiliations:** 1 School of Engineering and Digital Sciences, Nazarbayev University, Nur-Sultan 010000, Kazakhstan; akbota.sametova@nu.edu.kz (A.S.); sabit.kurmashev@nu.edu.kz (S.K.); zhashikbayeva@nu.edu.kz (Z.A.); aida.amantayeva@alumni.nu.edu.kz (A.A.); 2 Université Côte d’Azur, INPHYNI, CNRS UMR7010, Avenue Joseph Vallot, 06108 Nice, France; wilfried.blanc@inphyni.cnrs.fr; 3 Department of Chemistry, Nazarbayev University, 53 Kabanbay Batyr Avenue, Nur-Sultan 010000, Kazakhstan; timur.atabaev@nu.edu.kz; 4 National Laboratory Astana, Laboratory of Biosensors and Bioinstruments, Nur-Sultan 010000, Kazakhstan

**Keywords:** radiofrequency ablation, fiber-optic shape sensors, optical fiber sensor, distributed sensors, gold nanoparticles, biomedical sensors

## Abstract

In this work, we report the design of an optical fiber distributed sensing network for the 2-dimensional (2D) in situ thermal mapping of advanced methods for radiofrequency thermal ablation. The sensing system is based on six high-scattering MgO-doped optical fibers, interleaved by a scattering-level spatial multiplexing approach that allows simultaneous detection of each fiber location, in a 40 × 20 mm grid (7.8 mm^2^ pixel size). Radiofrequency ablation (RFA) was performed on bovine phantom, using a pristine approach and methods mediated by agarose and gold nanoparticles in order to enhance the ablation properties. The 2D sensors allow the detection of spatiotemporal patterns, evaluating the heating properties and investigating the repeatability. We observe that agarose-based ablation yields the widest ablated area in the best-case scenario, while gold nanoparticles-mediated ablation provides the best trade-off between the ablated area (53.0–65.1 mm^2^, 61.5 mm^2^ mean value) and repeatability.

## 1. Introduction

The role of minimally invasive cancer thermotherapies is substantially extended in the latest years, as clinical trends show strong progress toward methods with limited invasiveness and consistent repeatability in the replacement of more invasive methods such as surgical resection [1,2,3]. Methods based on thermal ablation find consistent progress as the technological advances in miniaturization of the devices [4], sensing [5], and real-time imaging [6] allow for achieving substantial clinical improvements while maintaining minimal invasiveness and percutaneous insertion of the surgical device.

Thermal ablation procedures accomplish a successful cancer treatment by transferring energy in the form of electromagnetic waves from a source placed outside of the patient into an applicator, percutaneously inserted into the tumor location [7] or externally placed [8]; the energy dissipated into the applicator is converted into thermal energy, resulting in a localized rise of temperature within the tumoral tissue that spreads from the applicator to the peripheral side. Temperature values over 60 °C result in a nearly instantaneous mortality of cancer cells, while cytotoxic effects are recorded for temperature values over 42 °C [9]; the overall effect is evaluated by the thermal dosimetry [10,11], which integrates the temperature over the exposure time.

Thermal ablation methods differ on the frequency *f* of the electromagnetic waves, and consequently on the type of applicator involved in the energy delivery [5]. The four main methods that involve thermal heating are based on: (1) radiofrequency ablation (RFA, *f*~450 kHz), which uses an electrical RF generator and a miniature applicator with an electrode mounted on a single tip [12] or multiple tips [13]; (2) high-intensity focused ultrasound (HIFU, *f*~1.2 MHz), which uses an ultrasound generator and an external transducer that focuses the incoming waves into the tissue, with a non-contact applicator that ablates the tissue by a combination of thermal effects and cavitation [14]; (3) microwave ablation (MWA, *f*~2.4 GHz), which uses a microwave generator and an applicator shaped as a transmission line and operating as a near-field antenna [15]; and (4) laser ablation (LA, *f*~300 THz), which uses a mid-power laser source either externally firing into the tissue surface [9] or coupled into a large-core optical fiber for in situ delivery [16].

Among these procedures, RFA is highly versatile, and achieves growing success rates in the treatment of solid tumors such as hepatocellular carcinoma [2,17], and spinal tumors [18]. An RFA needle can integrate multiple miniature electrodes [19], or a single electrode with a high contact surface for rapid thermotherapies [20]; some applicators incorporate micro-thermocouples for sensing [21]. Outside of cancer care, RFA can be applied in the treatment of cardiac arrhythmia [22], and in interventional pain management [23,24].

The main research trends aimed at the technological improvement of RFA point in two different directions. The first area of interest involves the use of the advances in material sciences to improve the heating efficacy. A critical factor for the cancer treatment via RFA relies on the changes of electrical impedance of the tissues as the temperature approaches the 100 °C value [5]; the vaporization of the inner part of the tissue causes the impedance to abruptly rise, and therefore only a small portion of the electrical power is dissipated onto the tissue. Under this condition, medical generators enter a “safe mode”, discontinuing the power supply and interrupting the RFA procedure [25]. In order to improve over this effect, both agarose and chitosan gels [26] have been employed in order to reduce the tissue impedance. In addition, gold and silver nanomaterials [27] have been used in combination with agarose with the purpose of improving the thermal delivery, and ultimately increasing the ablated region.

The second area of interest is the use of advanced biosensors for the detection of physical parameters in situ prior to the ablation (for diagnostic purposes) and during the thermal treatment. Optical fiber sensors serve this purpose much better than electrical or mechanical sensors [28], as they can incorporate biological [29] and biophysical sensing [30], as well as owning key properties in terms of biocompatibility, miniaturization of the footprint, disposability, response time, and spatial distribution.

Several optical fiber biosensors have been reported for this purpose. Evers et al. [31] reported fiber-optic biosensors for liver tissue identification, discriminating the healthy and tumoral tissues with a spectroscopic probe. Loyez et al. [29] reported a plasmonic tilted fiber Bragg grating with the capability of in situ detection of cytokeratin biomarkers for the identification of tumoral cells for following treatments. Tosi et al. [32] reported a fiber-optic dual pressure and temperature sensor for measuring the pressure in the proximity of the RFA tip during an ex vivo procedure. Several authors also reported the use of fiber Bragg gratings (FBGs) [12,33] and distributed sensors [34,35], for the temperature detection during hyperthermia, exploiting the spatial resolution of optical fiber sensors.

From an application standpoint, and from the point of view of controlling the thermal ablation process in real-time [36], temperature sensing plays the largest role in measuring the effectiveness of the procedure since thermal damage is largely depending upon the instantaneous temperature and also the variability of the electrical and thermal properties of the tissues prevents from obtaining ablation patterns with high repeatability in the experimental conditions. Densely arrayed FBG sensors [37], chirped FBGs [30], long period gratings [38,39], and distributed sensors, particularly when arranged into a multi-fiber grid-shaped arrangement [34], have the possibility of sub-centimeter spatially resolved thermal sensing. However, while distributed sensors interrogated via optical backscatter reflectometry [33] can use inexpensive single-mode fibers or high scattering fibers as sensors without any additional manufacturing, FBG sensors are more expensive as they require inscription of multiple devices into the fiber, making the system less compatible with a disposable use.

Prior works were performed on RFA using ferromagnetic nanoparticles [26], and silver nanoparticles with a green-oriented synthesis method [40], showing an increase of the treated region when nanoparticles are inserted in situ in a solution of agarose or chitosan gel. However, gold nanoparticles (AuNP) represent the most popular method to extend the performance of thermotherapies, as AuNP have been demonstrated in radiofrequency [41] and laser [42] ablation. AuNP have been investigated as a method for improving the thermal treatments, since they combine their properties of ease of synthesis and biocompatibility [43] with their advantageous electrothermal effects: lowering the impedance of the tissue at the electrode contact point, hence extending the duration of RFA [44], and improving the heating process at the peripheral side of the tumor [45,46], hence targeting a wider region. In addition, AuNP improves the drug delivery systems within cancer tissues, as shown in previous works [27,47,48].

In this work, we consolidate the design of a distributed fiber-optic sensing network for real-time, mini-invasive, and spatially resolved thermal detection for AuNP-mediated RFA; results are presented by comparing the thermal response of pristine ablation with AuNP-mediated ablation with two different density levels (1 and 4 mg/mL), and agarose-mediated RFA. The biosensing system is based on a network of six optical fibers having high scattering, arranged in a spatial division multiplexing setup. The positioning of the fibers in the tissue allows sensing over a 40 × 20 mm grid, with 7.8 mm^2^ pixel size. RFA experiments have been performed ex vivo on bovine phantom, in pristine mode, mediated by agarose, and finally mediated by Au nanoparticles in different densities. The resulting 2-dimensional (2D) thermal maps allow for the recording of the different heating patterns and spatiotemporal trends, and investigate the efficacy of each ablation process.

## 2. Materials and Methods

### 2.1. Experimental Setup

**Figure 1:**
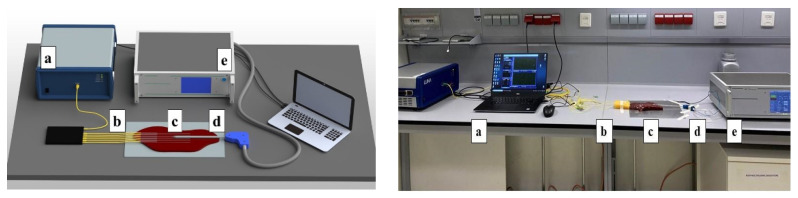
Schematic (**left**) and photographic view (**right**) of the radiofrequency ablation setup of the parenchymal tissue. The setup consists of: (a) OBR Luna 4600 with the computer used for data acquisition, (b) optical fibers, (c) bovine liver, (d) the RFA applicator, and (e) a hybrid RF/MWA generator used in RF mode.

The experimental setup of the thermal ablation procedure shown in Figure 1 comprises the following parts: (a) optical backscatter reflectometer (OBR, Luna 4600, Roanoke, VA, USA) with a computer used to collect and process the data; (b) distributed MgO nanoparticle-doped optical fibers spliced to single-mode fibers; (c) commercially obtained bovine liver; (d) RF applicator, with cylindrical shape (160 mm length, 3 mm diameter) and an active brass electrode on the conical tip of ~10 mm length; and (e) RF/MWA Hybrid Generator (LEANFA S.r.l., Ruvo di Puglia, Italy) that launches a 450-kHz radiofrequency signal to the applicator. The OBR is used in distributed sensing mode, measuring the frequency shift of each fiber signature within the network with millimeter-level spatial resolution [49]. Thermal ablation experiments employed six separate high-scattering optical fibers with different lengths plugged with the OBR Luna 4600 to measure temperature change during radiofrequency ablation.

### 2.2. Fiber Calibration

**Figure 2:**
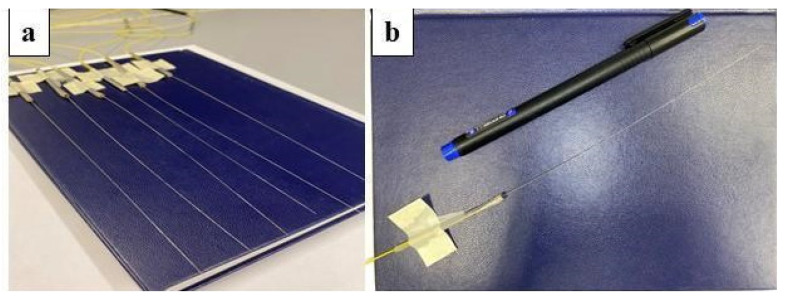
Photographic view of the MgO-doped fibers employed for temperature sensing. (**a**) Photos of the fibers displayed on a grid; (**b**) view of a single nanoparticle-doped optical fiber.

The presented work applied six Mg-silicate NPs fibers tagged M01 containing erbium in the core. The fabrication of M01 fiber includes solvents such as erbium (III) chloride hexahydrate and magnesium chloride in volumes of 10—4 mol/L and 0.1 mol/L, respectively [50]. Six MgO nanoparticle-doped optical fibers with the core diameter of 10 μm and the cladding diameter of 125 μm, matching the size and compound of single-mode glass fibers, have been used for the detection of the temperature change. Figure 2 shows the photographs of the used fibers.

**Figure 3:**
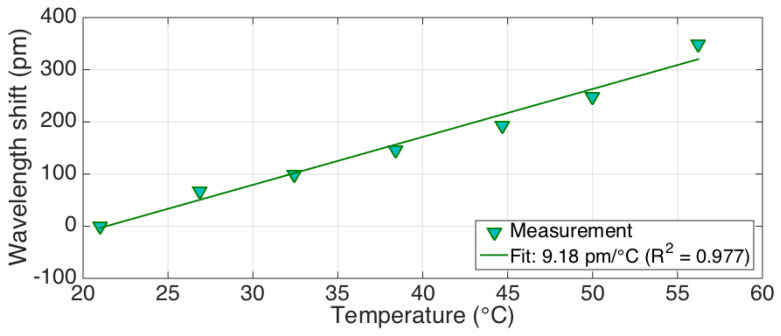
Calibration of the temperature coefficient of the optical fibers used for sensing.

We report in Figure 3 the thermal calibration of the fiber, obtained by exposing the fiber in a water bath heated by a thermal plate (IKA magnetic stirrer hot-plate, IKA-Werke Gmbh, Staufen, Germany), at temperatures ranging from 21 to 57 °C, and recording the temperature with a commercial fiber Bragg grating (Technica Optical Components LLC, Beijing, China) interrogated by an FBG (Fiber Bragg Grating) interrogator (si255 model, Micron Optics/Luna, Roanoke, VA, United States). The thermal coefficient is estimated as 9.18 pm/°C, which is very similar to FBG sensors working at 1550 nm (~10 pm/°C [32]).

### 2.3. Gold Nanoparticle Preparation

**Figure 4:**
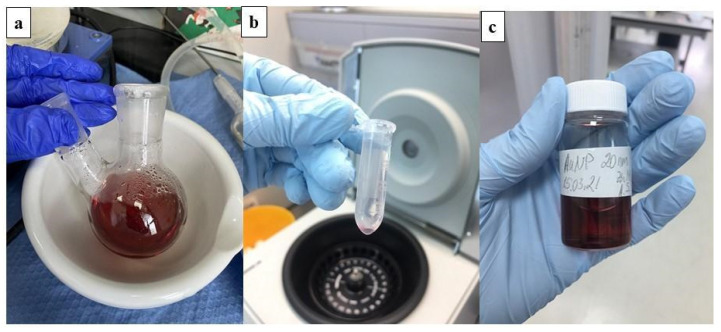
Gold nanoparticle cooling process (**a**) and after centrifuge (**b**,**c**).

**Figure 5:**
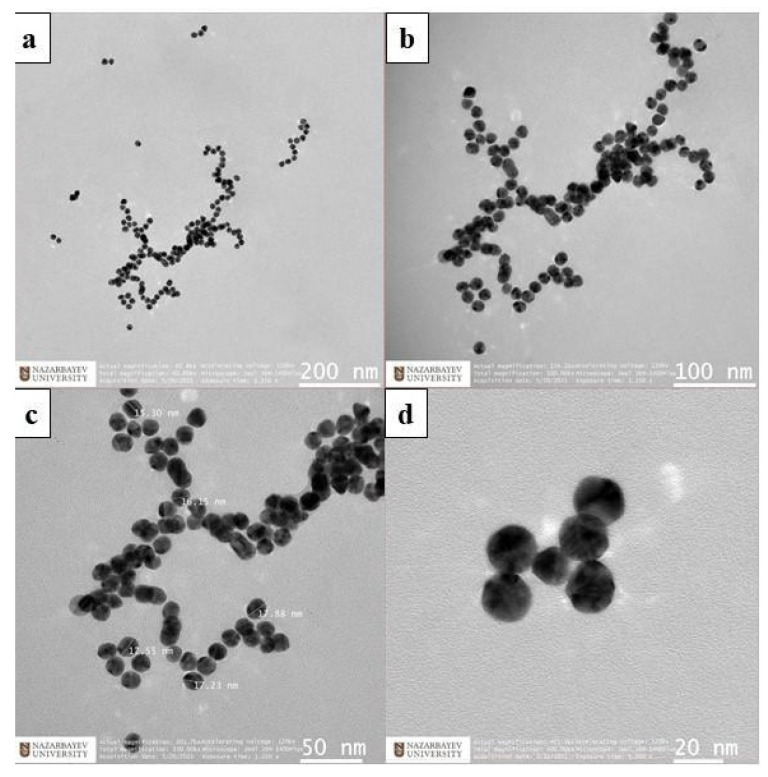
TEM images of AuNPs in different zooms: 200 nm (**a**), 100 nm (**b**), 50 nm (**c**), and 20 nm (**d**). The shape and size of AuNPs equivalent to 15–20 nm spheres in different zooms.

The presented study uses AuNPs of 15−20 nm size and in different densities introduced directly into the phantom. The synthesis of AuNPs was conducted using the citrate reduction method as presented by Turkevic et al. [51]. The 20 nm AuNPs were achieved by adding 2.0 mL of 34 mM trisodium citrate solution into a boiling solution, containing 0.5 mL of 1% hydrogen tetrachloroaurate (III) trihydrate and 50 mL of deionized water. The color of the solvent changed from bright yellow to dark violet in a few minutes and turned to ruby red after 15 min of stirring. The obtained solution was cooled at room temperature for the next 20 min and then cleaned by deionized water using a centrifuge operating at 15,000 rpm in 2-mL tubes (Figure 4). The size and shape of the synthesized GNPs were characterized using transmission electron microscopy (TEM) (Figure 5).

Agarose gel was prepared by mixing 1 g of agarose powder with Tris Acetate EDTA (TAE buffer) in a 50 mL volume and heating in the microwave until it dissolved completely. In order to obtain different densities of nanoparticles starting from 1 mg/mL to 4 mg/mL, AuNPs were dissolved in 0.2% agarose solution at a 1:1 to 1:4 ratio, respectively.

### 2.4. RF Ablation Experiments

**Figure 6:**
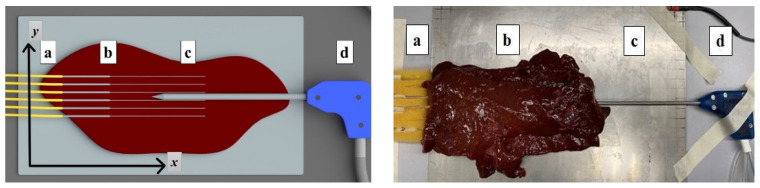
The scheme and photographic view fibers (a), bovine liver (b), negatively charged plate (c), and the applicator (d) in the vicinity.

Gold nanoparticles with different densities were prepared before thermal ablation experiments. The calibration of MgO-doped optical fibers was conducted before the experiments. Gold nanoparticles at different densities such as 1 mg/mL and 4 mg/mL were introduced ex vivo in the parenchymal tissue surface and around the applicator. The hybrid generator and reflectometer turned on at the same time: during the first phase, the tissue was ablated; then, the generator was automatically turned off by reaching the safe mode impedance value at 800 Ω, while the measurement was continued for the next 50 s. The applicator and fibers were positioned on opposite sides to each other on the *y*-axis; the distance between fibers was 4 mm on the *x*-axis and the tip of the applicator was placed between the third and fourth fibers (Figure 6). Thermal ablation experiments for each condition were repeated four times: pristine, pure agarose, gold nanoparticles with the density starting at 1 mg/mL, and gold nanoparticles with a density of 4 mg/mL to avoid contamination of fibers.

In the experiments, the applicator and fibers have been positioned manually by the operator in order to maintain the relative distance between the electrode tip and each fiber of the grid; agarose and agarose/AuNP were introduced after the fiber insertion, through a syringe. In order to preserve the fidelity of fiber insertion through the tissue, the fibers were inserted through the phantom, from one side; Figure 6a shows the cross-section of the tissue, while in Figure 6b, the photograph shows that in the actual experiments, the liver tissue was positioned around the fibers. Post-ablation photographs are displayed upon cutting the tissue in the cross-section.

**Figure 7:**
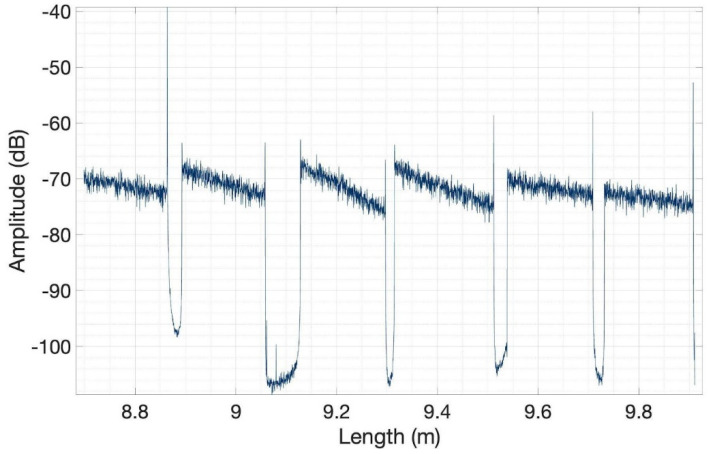
Backscattering trace, recording the Rayleigh backscattering intensity at every point along the fiber sensing network. The six regions with high intensity correspond to each nanoparticle-doped fiber span, while the low-intensity regions correspond to single-mode fibers used to distribute the signal to each sensing fiber.

MgO-doped optical fibers were spliced to single-mode fibers using a standard telecom splicer, resulting in a sensing system that detects the temperature change during radiofrequency ablation with 1-s speed. Figure 7 shows the resulting amplitude trace recorded on the OBR when all six fibers are connected, forming a scattering-level multiplexing network that allows simultaneous temperature recording on each location. The scattering gain of each nanoparticle-doped fiber is about 40 dB. The received data were first processed using a threshold algorithm which can identify sudden dips in signal amplitude and, thus, exact locations of all six fibers’ ends were discovered. Then, the peak temperature reading, which is in correspondence of the vertical coordinate of the applicator tip, was determined for each fiber and 20 data points around it were used for interpolation (using spline functions). Finally, interpolated domains were converted to thermal maps to accurately monitor the temperature change area.

## 3. Experimental Results

### 3.1. Thermal Maps

**Figure 8:**
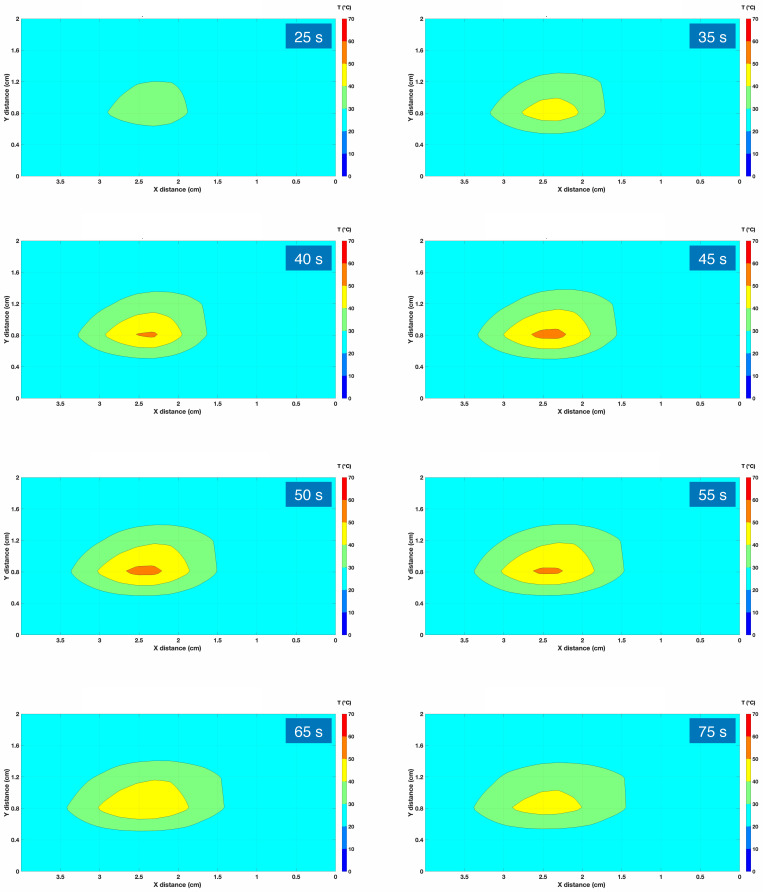
Thermal map recorded for an RFA experiment, using AuNP with a density of 4 mg/mL. The chart reports the data recorded on the xy plane (x = direction parallel to the RFA applicator and to the sensing fibers). The colorimetric map reported the isothermal curves, with 10 °C separation between each layer. Eight different maps are reported, during the heating phase (with times 25, 35, 40, 45, and 50 s from the RF generator power on), and during the cooling phase when the RF power is discontinued (55, 65, and 75 s elapsed time). The horizontal lines on each chart report the position of the six fibers.

We report in Figure 8 an example of a thermal map, recorded with the previously described setup for RFA ablation carried out with AuNP with a density of 4 mg/mL. Data are recorded on a xy grid with size 40 mm × 20 mm; the pixel of the grid is 2.5 mm (OBR spatial resolution after processing) × 5.0 mm (distance between each MgO-doped fiber), for a total of 102 sensing points (one sensor per each 7.8 mm^2^). Data reported in the figure show the isothermal curves, spacing the temperature data by 10 °C.

During the first part of the ablation, we observe a progressive heating as the temperature rises, peaking in correspondence to the active electrode. After 40 s, the inner temperature overcomes the 60 °C threshold, reaching the maximum extension after 50 s. At this point, the RF generator gets discontinued, causing the temperature to drop.

**Figure 9:**
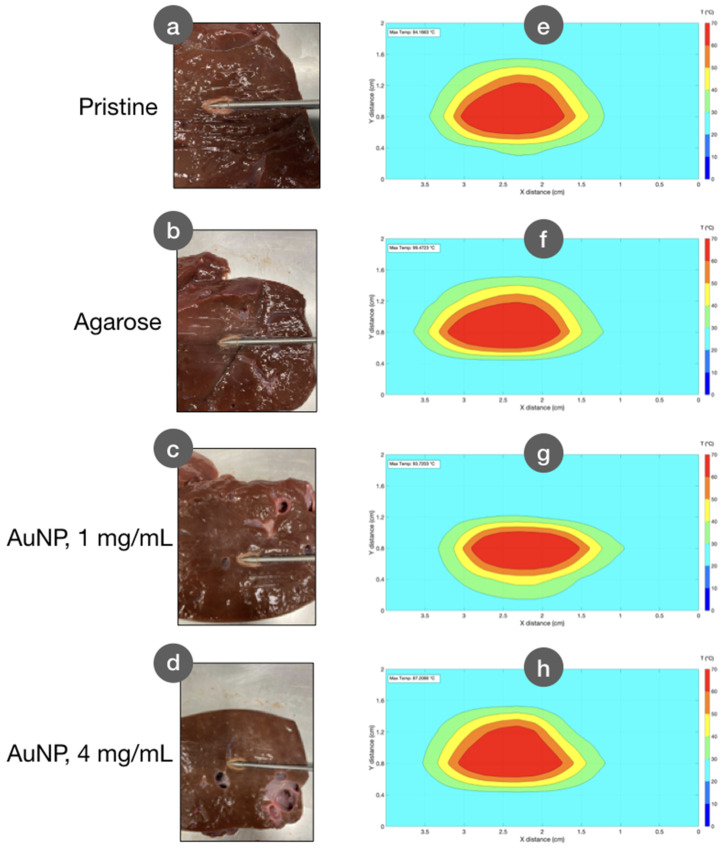
Thermal maps observed for different conditions, compared to the photograph of the ablated tissue. (**a**–**d**) Photographs of the tissue after RFA procedure; (**e**–**h**) thermal maps observed at the peak temperature condition. Experiments have been performed in pristine condition (**a**,**e**), with agarose gel inserted in the tissue (**b**,**f**), and with AuNP with densities of 1 mg/mL (**c**,**g**) and 4 mg/mL (**d**,**h**).

In Figure 9, we compare the thermal map observed at the peak temperature, in correspondence to the RF power discontinued from the generator. Thermal maps are showed in comparison with the pictures of the ablated tissue, for each different experiment: pristine ablation, agarose-mediated ablation, and AuNP-mediated ablation with densities of 1 mg/mL and 4 mg/mL. We observe that the experiments return a similar result in terms of the shape of the ablated region; AuNP-mediated RFA, however, results in a slightly elongated pattern of the isothermal curves.

**Figure 10:**
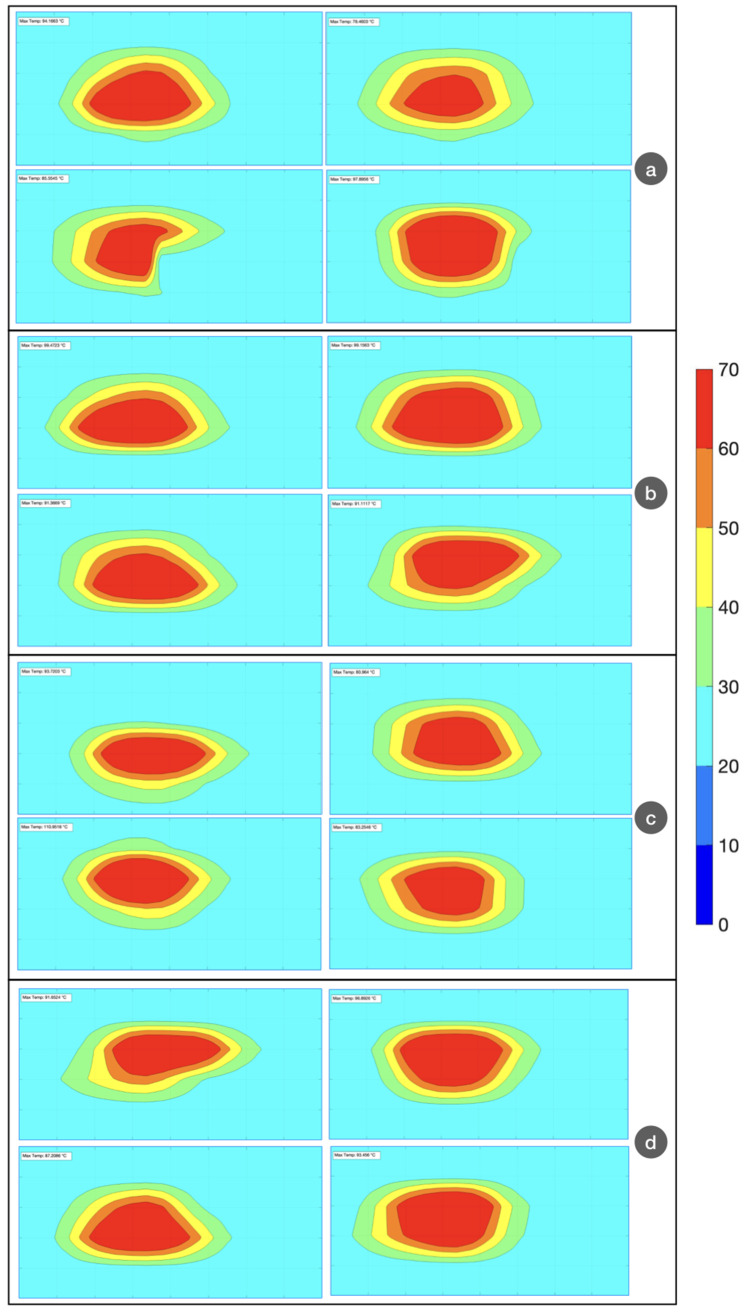
Evaluation of all thermal maps for four experiments, under each condition: (**a**) pristine; (**b**) agarose; (**c**) AuNP, 1 mg/mL; (**d**) AuNP, 4 mg/mL. The chart reports isothermal curves, according to the color bar on the right. Horizontal axis: x direction (4 cm range); vertical axis: y direction (2 cm range).

The experiments carried out during RFA are subjected to a high variability of results [27]. While the distribution of the nanoparticles in the tissue appears to play a role [40], the major source of variability is due to the different properties of the tissues: this is both attributable to the differences in electrical and thermal properties of the parenchyma, which also tend to increase in tumoral tissues [31], and also to the presence of blood vessels that prevent an efficient heat distribution [52]. In order to account for the repeatability of the ablation process, we performed four experiments for each ablation type, all using a similar phantom. The results are shown in Figure 10, where the thermal maps at the temperature peak are reported. We observe a difference in the shape and extension of the thermal zones: in particular, the AuNP-mediated isothermal curves appear to have a rounder contour, more similar to an elliptical shape; conversely, pristine ablation returns isothermal shapes characterized by more irregularities.

### 3.2. Maximum Temperature

**Figure 11:**
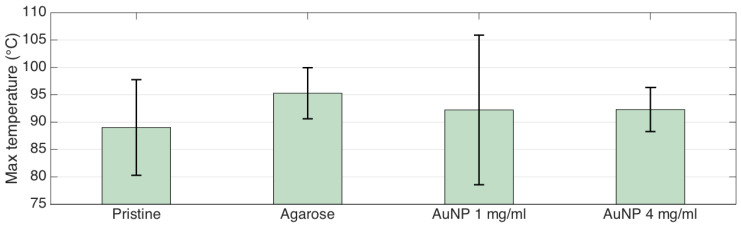
Peak temperature recorded during the RFA experiments under each condition; bars = average of four experiments; error bars = ±standard deviation.

The maximum temperature is an important indicator of the heating process occurring during RFA. According to Rhim et al. [53], temperature values exceeding 120 °C should be avoided, while the 80−100 °C peak temperature ensures the most efficient ablation process. Figure 11 shows the peak temperature recorded for all experiments, reporting both the average and the standard deviation. Pristine ablation shows an average temperature of 89.0 °C (the lowest value); whole agarose-mediated RFA shows 95.3 °C average (the highest value); and AuNP-mediated RFA returns similar temperature values (92.2 and 92.3 °C, respectively). AuNP-mediated RFA with 4 mg/mL shows the best repeatability of peak temperature (standard deviation = 4.0 °C), while AuNP-mediated ablation shows the worst repeatability (standard deviation = 13.7 °C).

**Figure 12:**
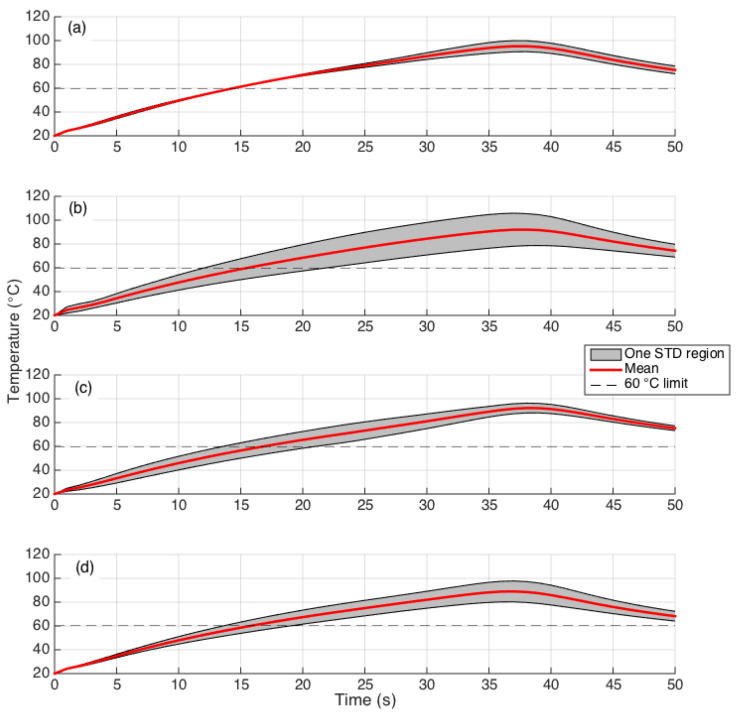
Temporal evolution of the maximum temperature over time, for each RFA ablation. The chart reports the results of four experiments, displaying the mean value (red curve) and the range containing ± one standard deviation (grey interval). RFA conditions: (**a**) agarose; (**b**) AuNP, 1 mg/mL; (**c**) AuNP, 4 mg/mL; (**d**) pristine.

The temporal evolution of the peak temperature shows insights on the heating process and the duration of the ablation procedure [54]. In Figure 12, the temporal trend of the peak temperature is reported for each experimental condition, for the first 50 s of the ablation process. We observe that the duration of the ablation process is similar for all conditions, about 37 s with a larger variability for the AuNP-mediated condition, 1 mg/mL and pristine ablation, and a smaller extension of the standard deviation region for the AuNP-mediated ablation, 4 mg/mL and agarose-mediated. Looking at the average trend, the agarose-mediated ablation reaches the 60 °C threshold in the fastest time (15 s), about 2 s faster than the pristine ablation.

### 3.3. Evaluation of Thermal Damage Regions

Thermal dosimetry estimates the amount of thermal damage induced by the ablation process [10]. The mortality rate of cancer cells is nearly instantaneous for temperature values higher than 60 °C, while no damage is induced below 42−44 °C depending on the type of tumor. For intermediate values, the mortality rate is a function of the temperature and the exposure time, and a common estimate is 52 °C for one minute of exposure. However, since RFA is a faster process with a duration of ~35 s, we can highlight three regions: (1) maximum thermal damage (temperature >60 °C), where the mortality rate is almost ideal; (2) cytotoxic region (temperature between 42 °C and 60 °C), where temperature induces a partial damage of the tissue; and (3) safe region (temperature below 42 °C), unaffected by RFA. The possibility of drawing isothermal curves at the moment of maximum heating determines the width of each region.

**Figure 13:**
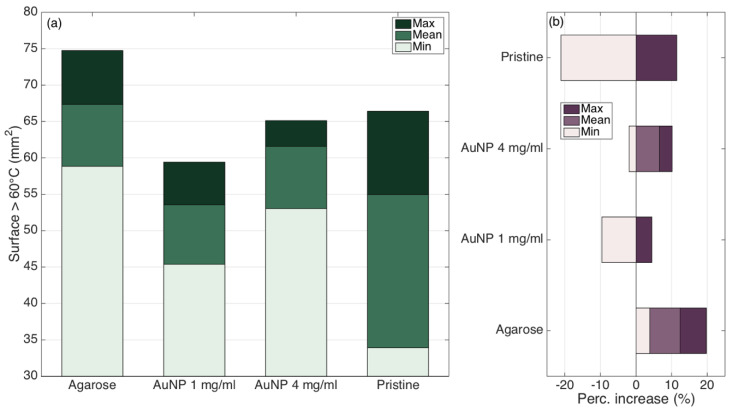
Quantification of the thermal damage region (temperature >60 °C) for each experimental condition. (**a**) Evaluation of the surface exposed to temperature exceeding 60 °C at the maximum ablation temperature; bar charts show the maximum (dark), minimum (bright), and mean (intermediate) values of the areas, over four experiments for each RFA condition. (**b**) Percentual increase or decrease for the ablated surface, with respect to the reference condition (pristine RFA, mean surface).

Figure 13 shows the quantification of the thermal damage condition. Pristine ablation shows an ablated surface of 55.0 mm^2^ (mean value), and has the widest extension (33.9–66.4 mm^2^). Agarose-mediated RFA shows the highest extension of the ablated region (67.3 mm^2^ mean value, 74.7 mm^2^ maximum). On the other side, the AuNP-mediated ablation with 4 mg/mL density shows the best trade-off between amount of ablated tissue (61.5 mm^2^ mean value) and repeatability (53.0–65.1 mm^2^ minimum−maximum values). The percentual indicators displayed on the right chart show the quantification of the increase or decrease of ablated tissue with respect to pristine ablation (mean value): it shows that pristine ablation has the worst amount of ablated tissue and the widest repeatability range, while agarose-mediated RFA can ablate up to 19.8% more of tissue.

**Figure 14:**
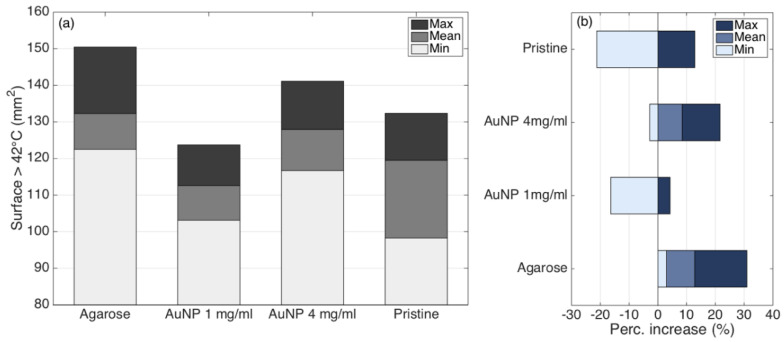
Quantification of the cytotoxic region (temperatures >42 °C) for each experimental condition. (**a**) Evaluation of the surface exposed to temperatures exceeding 42 °C at the maximum ablation temperature; bar charts show the maximum (dark), minimum (bright), and mean (intermediate) values of the areas, over four experiments for each RFA condition. (**b**) Percentual increase or decrease for the cytotoxic surface, with respect to the reference condition (pristine RFA, mean surface).

In Figure 14, the cytotoxicity regions are shown, reporting the areas of the tissue exposed to temperatures >42 °C. We observe a similar trend with respect to the thermal damage results, with agarose performing the widest region of damage (mean value 132.3 mm^2^) but with the widest range, and AuNP-mediated nanoparticles with the higher density to represent the best trade-off between amount of thermal damage (mean value 127.9 mm^2^, +8.5% with respect to pristine conditions) and repeatability (116.7–141.1 mm^2^ minimum−maximum values).

## 4. Discussion

The display of results shows the effectiveness of the ablation method, and the importance of the in-situ sensing device for the real-time detection of temperature patterns. A first point of discussion involves the efficacy of temperature sensing in the context of thermal ablation. As highlighted in Figure 10, the lack of repeatability of RFA requires a precise thermal control in situ: distributed sensing in this case is a very effective technology, as it ensures not only rapid sensing with accurate detection (about 1 pm uncertainty, corresponding to ~0.1 °C), but the entire sensing network is designed for the possible in vivo use, since the fibers have miniature form factor, biocompatibility, and the sensing mechanism is robust with respect to the catheterization options [28] and possible strains occurring during the insertion [55]. The proposed sensing network has the potential of being much more cost-effective than FBG arrays, in terms of disposable use. It is in fact essential to ensure that the cost of the whole sensing device that complements the RFA device is a fraction of the applicator itself, in order to ensure an affordable treatment; while FBG arrays have a cost of a few hundreds of dollars per array, the MgO-doped fiber can be fabricated with the same technology in terms of doping, preforming, and drawing of SMF fibers that cost a few dollar cents per meter (about $0.08/m in current markets). Additionally, MgO-doped fibers can be spooled and spliced to SMF fibers using standard splicers, without the need to develop new splicing methods. In addition, FBG sensors encode the sensing in a specific location, and therefore they are both limited by the grating length and misalignment of each grating position [12]. Distributed sensing, on the other hand, can improve the spatial resolution down to 10 μm (theoretical value of the OBR [56]), and since the entire fiber acts as a sensor, it is possible to align thermal maps using signal processing methods or other artefacts [57]. On the other hand, distributed sensing provides a much more detailed detection with respect to thermocouples, even in miniaturized formats [58], or fluorescence-based detectors [59], as these methods are intended for single-point sensing while the proposed method can resolve several tens of sensors per each cm^2^.

Regarding the thermal ablation process, the analysis of cytotoxicity and thermal damage levels shows that the RFA process improves when using agarose and Au nanoparticles with the appropriate density. While agarose-mediated RFA achieves the best results in terms of heat delivery, guaranteeing the largest thermal ablation results, the use of AuNP shows the best compromise between the width of the ablated area and the need for a repeatable process. As validated by experiments, by using 4 mg/mL density, the best results can be achieved. The possibility of extending the ablated region is an important asset in advancing RFA to treat larger types of cancer, particularly for hepatic tumors [60].

## 5. Conclusions

In conclusion, we reported the use of a distributed fiber-optic sensing network optimized for the detection of thermal patterns in RFA procedure, mediated by the use of agarose and gold nanoparticles for the improvement of the heat delivery. The fiber-optic sensing network is formed by a set of six MgO-NP fibers, deployed over a grid of 40 × 20 mm, forming 102 sensing points spanning the spatial resolution of the interrogator on the horizontal axis and the spacing between each fiber on the vertical axis.

Several experiments of RFA were performed, in pristine condition as well as mediated by agarose and AuNP (1 and 4 mg/mL density). Thermal maps and isothermal representation allow precise identification of the ablated region, determining the area exposed to the ideal thermal damage (60 °C and above) and cytotoxicity levels for partial mortality (between 42 and 60 °C). Experimental results show that agarose-mediated thermal ablation yields the widest treated area, but with low repeatability; on the other hand, AuNP-mediated ablation using 4 mg/mL density provides the best trade-off between efficacy of ablation and repeatability.

Future work will further consolidate the RFA applicator, the on-board sensors for real-time detection, and the nanoparticle delivery into a single device, scaling up the possibility of using this arrangement in clinical settlements.

## Data Availability

Data presented in this work are not publicly available at this time, but can be obtained upon reasonable request from the authors.
